# Solid-Phase Microextraction and Related Techniques in Bioanalysis

**DOI:** 10.3390/molecules28062467

**Published:** 2023-03-08

**Authors:** Hiroyuki Kataoka

**Affiliations:** Laboratory of Applied Analytical Chemistry, School of Pharmacy, Shujitsu University, Nishigawara, Okayama 703-8516, Japan; hkataoka@shujitsu.ac.jp; Tel.: +81-86-271-8342

Living organisms, such as microorganisms, plants and animals, are composed of complex constituents, which may include bioactive components that maintain their functions. In addition, these organisms may contain harmful external contaminants. Bioanalysis of these endogenous substances, metabolites and contaminant poisons is important in analyses of biological functions, metabolomics, forensic toxicology, patient diagnosis and the biomonitoring of human exposure to hazardous chemicals. In these analyses, sample preparation is essential for the isolation and concentration of target analytes from complex biological matrices, including serum/plasma, urine, saliva, hair, breath and tissue. However, preparation processes are time-consuming, labor-intensive and error-prone, and they markedly influence the reliability and accuracy of determining molecules of interest. Thus, effective sample preparation techniques and their integration with analytical methods have become a prominent research topic.

Solid-phase microextraction (SPME) is a simple and convenient sample preparation technique that enables automation, miniaturization, high-throughput performance and online coupling with analytical instruments. Moreover, SPME reduces analysis times, as well as solvent and disposal costs. Since SPME was first introduced in the early 1990s [1], more robust fiber assemblies and coatings with higher extraction efficiencies, selectivity and, stability have become commercially available. Furthermore, new geometries have been designed for extraction as alternatives to fibers, such as capillary tubes, magnetic stir bars or thin films; moreover, novel intelligent polymer coatings with great sorption capacity or good selectivity have been developed for use as extraction phases.

This Special Issue, entitled “Solid-Phase Microextraction and Related Techniques in Bioanalysis”, consists of 14 original, peer-reviewed papers written by research groups worldwide. The topics covered include headspace fiber SPME (HS-SPME) gas chromatography–mass spectrometry (GC-MS) [2,3,4,5,6,7,8], HS-in-needle ME (HS-INME)-GC-MS [9], thin film SPME (TF-SPME) liquid chromatography–tandem mass spectrometry (LC-MS/MS) [10], magnetic solid-phase extraction (MSPE) LC-MS/MS [11], in-tube SPME (IT-SPME) LC-MS/MS [12,13,14] and IT-SPME LC-UV [15]. The samples analyzed include a wide range of plant-derived volatile organic compounds [2,3,4,5,6,7]; body odor from the skin [8,9]; metabolites from urine [10], plasma [11] and saliva [12] samples; biomarkers of exposure to tobacco smoke in hair [13,14]; and environmental estrogens in water [15]. An overview of these papers is provided below.

Profiles of volatile organic compounds (VOCs) emitted by plants were analyzed using HS-SPME GC-MS [2,3,4]. The tastes and aromas released during fruit ripening play an important role in the identification of cultivars and the quality of fruits and their products, including their characteristic flavors. Fragrance components include a variety of low-molecular-weight VOCs, such as alcohols, esters, acids, aldehydes, ketones, aliphatic and aromatic esters, terpenes, hydrocarbons, phenols and sulfur compounds. An HS-SPME GC-MS analysis of the volatility profile in nine species of rumberry (*Myrciaria floribunda*) fruits identified 36 VOCs, among which the sesquiterpenes caryophyllene and γ-selinene were found to be flavor-determining components [2]. HS-SPME GC-MS conditions were optimized to extract, detect and quantify volatile components from the pulp of *Eugenia klotzschiana* O. Berg, a landrace of the Cerrado biome with important nutritional value, and its aroma was found to be composed of 38 sesquiterpene and monoterpene compounds [3]. HS-SPME GC-MS also identified 22 VOCs in the essential oil of dioecious aroeira seeds of *Myracrodruon urundeuva* using HS-SPME GC-MS, and their anti-inflammatory properties were analyzed using a chemoinformatics approach [4]. A combination of HS-SPME GC-MS and multivariate statistical analysis was used to isolate and identify VOCs in *Herpetospermum pedunculosum*, a dioecious plant that has been used as a traditional Tibetan medicine for the treatment of hepatobiliary diseases [5]. These analyses showed that the levels of nine VOCs, including isoamyl alcohol, (Z)-3-methylbutanal oxime and 1-nitropentane, differed significantly in female and male flower buds.

The HS-SPME GC-MS method was also used to identify damage to crops caused by pests [6,7]. Because stored crops can be affected by pests and parasites, the prevention of damage and maintenance of quality parameters over time are important for ensuring global food security. Rapid and appropriate methods of sampling, pest detection and data analysis are therefore required to control crop quality in real time. VOCs from crops and pests can serve as biomarkers for monitoring pest damage. For example, HS-SPME GC-MS identified wheat- and insect-specific VOCs, including benzoquinone homologues, released by three stored grain pests, suggesting that these compounds can act as biomarkers of crop damage [6]. Similarly, HS-SPME GC-MS was used to compare VOCs from cabbage plants that were and were not infected with the green peach aphid *Myzus persicae* [7]. Among the 28 VOCs detected in these plants, several, including propane; 2-methoxy; alpha- and beta pinene; myrcene; 1-hexanone; 5-methyl-1-phenyl-; limonene; decane; γ-terpinen and heptane; and 2,4,4-trimethyl propane, were more abundant in infected plants; this indicated that these compounds were responsible for aphid attraction, and therefore, useful in screening for *M. persicae* infection [7].

The odors and emanations released from the human body can provide important information about an individual’s health status and the presence or absence of disease. Among the various VOCs emitted from human skin, trans-2-nonenal, benzothiazole, hexyl salicylate, α-hexyl cinnamaldehyde, and isopropyl palmitate are important indicators of the degree of aging [9]. Because these compounds often emanate from the body’s surface in very small quantities, simple sampling and sensitive analytical methods are required. Two methods are available for sampling body odor using SPME: an in vivo method, in which SPME fibers are placed on exposed skin, and an in vitro method, in which SPME fibers indirectly extract body odor from a cotton swab or T-shirt. For example, an in vitro method for the HS-SPME GC-MS analysis of trans-2-nonenal consisted of wiping body odor from the skin’s surface with gauze, followed by an analysis of changes in body odor in response to lifestyle changes [8]. In contrast, in vitro and in vivo HS-INME GC-MS analyses of body odor VOCs involved solid dynamic microextraction using the adsorbent-coated inner wall of the needle of a gas-tight syringe, or an adsorbent-coated stainless steel wire inserted into the needle [9].

Cystoscopy is an invasive and uncomfortable procedure for patients with bladder cancer, and clinical tests, such as cytology of urine sediment, have low sensitivity and specificity in the monitoring of early-stage bladder cancer. The untargeted metabolomic/metabolomic profiling of biological fluids may be a more effective and less invasive approach to identifying biomarkers of bladder cancer, along with the development of new biomarker-based diagnostic tools. For example, the metabolomic profiling of urine samples from bladder cancer patients and healthy controls has been performed using high throughput TF-SPME LC-MS [10].

Starting with cholesterol, steroid hormones are biosynthesized by various enzymes in the adrenal cortex, gonads and brain, and are subsequently metabolized via phase I oxidation and reduction reactions and phase II conjugation reactions. However, these biosynthetic and metabolic pathways are complex, and their molecular roles are not fully understood. The analysis of steroid hormones and their metabolites is important for elucidating biological regulatory mechanisms and diagnosing diseases related to them. One method of achieving this is dispersive MSPE, in which a magnetic sorbent is dispersed in a sample solution, such as plasma, the solution stirred to extract the compounds of interest, and the extracted compounds eluted from the magnetic sorbent are used in MSPE LC-MS/MS analysis of glucocorticoids, estrogens, progestogens and androgens [11]. Furthermore, an automated analysis system that couples on-line IT-SPME and LC-MS/MS was constructed using an open-tube fused silica capillary with a coated inner surface as the extraction device, enabling simultaneous selective and sensitive analysis of the metabolism of sulfated steroids in saliva samples [12].

Tobacco smoking and exposure to environmental tobacco smoke are considered risk factors for cancers, cardiovascular diseases and respiratory disease, and these health effects have become a serious problem. Tobacco-related compounds, such as nicotine, its metabolite cotinine and tobacco-specific nitrosamines, have been used as biomarkers of exposure to tobacco smoke. Levels of nicotine and cotinine in the hair of non-smokers were analyzed as biomarkers of exposure to tobacco smoke using IT-SPME LC-MS/MS, in order to determine the risks of passive smoking in different lifestyle environments [13]. In addition, online IT-SPME LC–MS/MS was used to simultaneously measure the content of five tobacco-specific nitrosamines in hair as biomarkers of exposure to tobacco smoke [14].

Finally, an IT-SPME method using extraction tubes filled with hydrophilic, superhydrophilic, superhydrophobic and UV-irradiated superhydrophilic Ti wires as sorbents was used for on-line IT-SPME LC-UV analysis of six estrogen-like hormones in water samples [15].

## Data Availability

Not applicable.

## References

[1] Arthur C.L., Pawliszyn J. (1990). Solid phase microextraction with thermal desorption using fused silica optical fibers. Anal. Chem..

[2] García Y.M., Ramos A.L.C.C., de Paula A.C.C.F.F., do Nascimento M.H., Augusti R., de Araújo R.L.B., de Lemos E.E.P., Melo J.O.F. (2021). Chemical Physical Characterization and Profile of Fruit Volatile Compounds from Different Accesses of *Myrciaria floribunda* (H. West Ex Wild.) O. Berg through Polyacrylate Fiber. Molecules.

[3] Mariano A.P.X., Ramos A.L.C.C., de Oliveira A.H., García Y.M., de Paula A.C.C.F.F., Silva M.R., Augusti R., de Araújo R.L.B., Melo J.O.F. (2022). Optimization of Extraction Conditions and Characterization of Volatile Organic Compounds of *Eugenia klotzschiana O*. Berg Fruit Pulp. Molecules.

[4] Figueiredo Y.G., Corrêa E.A., de Oliveira A.H., Mazzinghy A.C.d.C., Mendonça H.d.O.P., Lobo Y.J.G., García Y.M., Gouvêia M.A.d.S., de Paula A.C.C.F.F., Augusti R. (2022). Profile of *Myracrodruon urundeuva* Volatile Compounds Ease of Extraction and Biodegradability and In Silico Evaluation of Their Interactions with COX-1 and iNOS. Molecules.

[5] Liu Z., Fang Y., Wu C., Hai X., Xu B., Li Z., Song P., Wang H., Chao Z. (2022). The Difference of Volatile Compounds in Female and Male Buds of *Herpetospermum pedunculosum* Based on HS-SPME-GC-MS and Multivariate Statistical Analysis. Molecules.

[6] Cai L., Macfadyen S., Hua B., Zhang H., Wei Xu W., Ren Y. (2022). Identification of Biomarker Volatile Organic Compounds Released by Three Stored-Grain Insect Pests in Wheat. Molecules.

[7] Ahmed Q., Agarwal M., Alobaidi R., Haochuan Zhang H., Ren Y. (2022). Response of Aphid Parasitoids to Volatile Organic Compounds from Undamaged and Infested *Brassica oleracea* with *Myzus persicae*. Molecules.

[8] Saito K., Tokorodani Y., Sakamoto C., Kataoka H. (2021). Headspace Solid-Phase Microextraction/Gas Chromatography–Mass Spectrometry for the Determination of 2-Nonenal and Its Application to Body Odor Analysis. Molecules.

[9] Kim S., Bae S. (2022). In Vitro and In Vivo Human Body Odor Analysis Method Using GO:PANI/ZNRs/ZIF−8 Adsorbent Followed by GC/MS. Molecules.

[10] Łuczykowski K., Warmuzińska N., Operacz S., Stryjak I., Bogusiewicz J., Jacyna J., Wawrzyniak R., Struck-Lewicka W., Markuszewski M.J., Bojko B. (2021). Metabolic Evaluation of Urine from Patients Diagnosed with High Grade (HG) Bladder Cancer by SPME-LC-MS Method. Molecules.

[11] Speltini A., Merlo F., Maraschi F., Marrubini G., Anna Faravelli A., Profumo A. (2021). Magnetic Micro-Solid-Phase Extraction Using a Novel Carbon-Based Composite Coupled with HPLC–MS/MS for Steroid Multiclass Determination in Human Plasma. Molecules.

[12] Kataoka H., Nakayama D. (2022). Online In-Tube Solid-Phase Microextraction Coupled with Liquid Chromatography–Tandem Mass Spectrometry for Automated Analysis of Four Sulfated Steroid Metabolites in Saliva Samples. Molecules.

[13] Kataoka H., Kaji S., Moai M. (2021). Risk Assessment of Passive Smoking Based on Analysis of Hair Nicotine and Cotinine as Exposure Biomarkers by In-Tube Solid-Phase Microextraction Coupled On-Line to LC-MS/MS. Molecules.

[14] Ishizaki A., Kataoka H. (2021). Online In-Tube Solid-Phase Microextraction Coupled to Liquid Chromatography–Tandem Mass Spectrometry for the Determination of Tobacco-Specific Nitrosamines in Hair Samples. Molecules.

[15] Zhang Y., Wang N., Lu Z., Chen N., Chengxing Cui C., Chen X. (2022). Smart Titanium Wire Used for the Evaluation of Hydrophobic/Hydrophilic Interaction by In-Tube Solid Phase Microextraction. Molecules.

